# Association of sclerostin with cardiovascular events and mortality in dialysis patients

**DOI:** 10.1080/0886022X.2020.1741386

**Published:** 2020-03-26

**Authors:** Yun Zou, Min Yang, Jiao Wang, Li Cui, Zhenxing Jiang, Jiule Ding, Min Li, Hua Zhou

**Affiliations:** a Department of Nephrology, The Third Affiliated Hospital of Soochow University, Changzhou, China; b Changzhou Center for Animal Disease Control and Prevention, Changzhou, China; c Department of Urology, The Third Affiliated Hospital of Soochow University, Changzhou, China; d Department of Medical Imaging, The Third Affiliated Hospital of Soochow University, Changzhou, China

**Keywords:** Cardiovascular diseases, dialysis, mortality, sclerostin, vascular calcification

## Abstract

**Introduction:**

Sclerostin has been reported to be a novel biomarker associated with the bone-vascular axis. In this study, we determined the relationships between serum sclerostin and all-cause mortality, the prevalence of cardiovascular events (CVEs), and coronary artery calcifications (CACs) in dialysis patients.

**Methods:**

A total of 165 dialysis patients (84 hemodialysis [HD] and 81 peritoneal dialysis [PD]) were enrolled in this study. We performed multivariable linear regression analysis to test the relationships between serum sclerostin levels and demographics and clinical parameters. We also performed Cox proportional hazard regression analysis to determine independent predictors of overall survival and CVEs.

**Results:**

The median serum sclerostin level was 250.9 pg/mL in dialysis patients. Kaplan–Meier analysis showed that both overall and CVE-free survival rates were significantly lower in the high serum sclerostin group (serum sclerostin level >250.9 pg/mL) compared to the low serum sclerostin group (serum sclerostin level ≤250.9 pg/mL) in patients with PD (*p* < 0.05). In patients with HD, only CVE-free survival rates notably declined in the high serum sclerostin group compared to the low serum sclerostin group (*p* = 0.029). However, serum sclerostin level was only an independent predictor of all-cause mortality and CVEs in patients with PD after adjusting for confounding factors (*p* < 0.05), and therefore was not an independent predictor for patients with HD (*p* > 0.05).

**Conclusions:**

A low serum sclerostin was associated with better overall survival and lower prevalence of CVEs in patients with PD, but had no relationships in patients with HD. We found that serum sclerostin level was not correlated with CACs in either patients with HD or PD.

## Introduction

Vascular calcifications (VCs) are a common complication and strong risk factor for cardiovascular morbidity and mortality in patients with end-stage renal disease (ESRD) [1]. The prevalence of VCs in ESRD patients is much higher than in the general population [2]. Coronary artery calcifications (CACs), especially the extent of CACs, are an independent predictor of cardiovascular events (CVEs) [3]. Previous studies have shown that CACs are associated with increased mortality in dialysis patients [4]. The transformation of vascular smooth muscle cells (VSMCs) to an osteoblastic phenotype is involved in the pathogenesis of ESRD-mediated VCs [5]. Several bone metabolism biomarkers, such as osteopontin [6], osteoprotegerin [7,8], and alkaline phosphatase (AKP) [9], have been shown to participate in VCs. Recently, sclerostin, an osteocyte-derived novel candidate protein, has attracted interest in the research community. Sclerostin is a major antagonist of the Wnt/β-catenin pathway that down-regulates bone metabolism [10]. Furthermore, there is accumulating evidence that the Wnt/β-catenin pathway is involved in bone-vascular cross-talk [11,12]. Therefore, these discoveries have prompted some to speculate that sclerostin may also play an extra-skeletal role in VCs [13]. Previous studies have shown a positive or negative relationship, as well as no correlation, between circulating sclerostin levels and VCs across different populations, and such inconsistent results have also been reported between circulating sclerostin levels and mortality [14–18]. Thus, the potential role of sclerostin in mortality, CVEs, and VCs warrants further study.

In this study, we determined the prognostic role of serum sclerostin levels in patients with hemodialysis (HD) and peritoneal dialysis (PD) in a prospective single center study. We investigated the associations between serum sclerostin levels and all-cause mortality, CVEs, and CACs in dialysis patients, as assessed by chest computed tomography (CT).

## Material and methods

### Study population

This prospective observational study was conducted in the Department of Nephrology of the Third Affiliated Hospital of Soochow University (Changzhou, China) from January 2012 to December 2014. All patients who received routine HD or PD for at least three months were recruited in the study and followed up until June 2016. Patients with hemodiafiltration, active infections or malignancies were excluded from the study. The study protocol conformed with the guidelines set forth by the Declaration of Helsinki. Written informed consent was obtained from all participants prior to enrollment in the study. The study was approved by the Ethics Committee of the Third Affiliated Hospital of Soochow University, China (registration number 24/2014).

### Study design

Anonymized data on demographics and clinical characteristics were collected at the time of enrollment. CVEs history was recorded, which was defined as coronary heart disease, heart failure, stroke, and peripheral arterial disease. Fasting blood samples were obtained prior to mid-week dialysis sessions for patients with HD. Routine biochemical tests, such as serum hemoglobin (Sysmex XN-9000^™^; Japan), creatinine, blood urea nitrogen (BUN), uric acid, calcium, phosphate, AKP, albumin, total cholesterol, triglycerides, low-density lipoprotein (LDL)-cholesterol, high-density lipoprotein (HDL)-cholesterol, C-reactive protein (CRP) [Beckman Coulter’s UniCel AU5800 immunoassay systems; USA], and intact parathyroid hormone (iPTH) [Beckman Coulter’s UniCel DxI 800 immunoassay systems; USA] were measured using standard laboratory methods. Residual renal function (RRF) was determined by Cystatin C formula [19]. Serum sclerostin levels were measured by commercially available enzyme-linked immunosorbent assay (ELISA) (R&D, USA) according to the manufacturer’s instructions. The intra- and inter-assay precision of the sclerostin ELISAs kits were 5.7% and 8.2%, respectively. The 5 mm chest CT (without ECG gating) was completed within three months of enrollment. The results were quantified automatically by the built in CT-software using the Agatston scoring method [20]. Briefly, a score for each region of interest was calculated by multiplying the density score and volume score. The threshold for a calcific lesion was set at a computed tomographic density of 130 Hounsfield units having an area ≥ 1 mm^2^.

### Primary end points

The primary end points were all-cause mortality and CVEs during the follow-up period. CVEs were defined as the incidence of coronary heart disease, heart failure, stroke, or peripheral arterial disease.

Patients were censored on kidney transplantation, loss of follow-up or death. Patients who have a switch of dialysis modality or transfer to other center were still followed for all-cause mortality and CVEs. Patient status was recorded at the end of the study (June 2016).

### Statistical analyses

All statistical analyses were conducted with Statistical Package for the Social Sciences (SPSS) software program, version 19.0 (SPSS, Chicago, IL, USA). All data were first checked for normality of distribution using the Kolmogorov–Smirnov test of normality. Normally distributed data were represented as the mean ± standard deviation. Non-normally distributed data were represented as the median (inter-quartile range [IQR]). The Mann–Whitney test was used for non-normally distributed continuous variables. Student’s *t* test was used to test for differences between groups for normally distributed continuous variables. The chi-square test was used to evaluate differences in prevalence. The Spearman correlation coefficient was used to test relationships between serum sclerostin levels and demographics as well as clinical parameters. A multivariable linear regression model was developed to identify the factors associated with serum sclerostin levels. Significant variables in the Spearman correlation analysis (*p* < 0.1) were included into the multivariable linear regression model. The Kaplan–Meier method was used to estimate the cumulative survival rate and new onset CVEs in dialysis patients with serum sclerostin levels above and below the median. Differences in survival curves were evaluated using the log-rank test. The independent relationship between serum sclerostin levels and the incidence rate of all-cause mortality and CVEs was investigated by univariate and multivariate Cox regression analyses. In multivariate analyses, models were adjusted for demographic variables (age and gender; model 1), Framingham cardiovascular risk factors (age, gender, smoking, diabetes status, systolic blood pressure [BP], and total and HDL-cholesterol; model 2), factors associated with mortality in patients with chronic kidney disease (CKD) (age, gender, CRP, phosphate, BMI, and albumin; model 3), and confounding factors associated with mortality and new onset CVEs in univariate analysis together with age and gender (model 4). A value of *p* < 0.05 was considered statistically significant.

## Results

### Baseline characteristics of the study subjects according to dialysis modality

A total of 165 dialysis patients (84 patients with HD and 81 patients with PD) were enrolled in this study. During the follow-up period, 11 patients underwent kidney transplantation, 2 patients were lost to follow-up, and 3 patients transferred to other center. The detailed baseline characteristics of both HD and PD groups are summarized in Table 1. Age, prevalence of diabetes, serum AKP, albumin, and iPTH were significantly higher in patients with HD compared to PD patients (*p* < 0.05). However, systolic BP, diastolic BP, serum creatinine, and corrected calcium were significantly lower in patients with HD compared to PD patients (*p* < 0.05).

**Table 1.** Demographic and biochemical characteristics of the 165 dialysis patients.

Characteristics	All patients (*n* = 165)	HD (*n* = 84)	PD (*n* = 81)	*p* Value
Age, year	56.5 ± 15.6	60.3 ± 15.2	52.5 ± 15.1	0.001
Male gender, *n*%	92 (55.8%)	48 (57.1%)	44 (54.3%)	0.715
Dialysis duration, months	24.5 (10.9, 48.7)	21.3 (10.4, 43.0)	27.9 (11.4, 56.3)	0.176
Diabetes, *n*%	48 (29.1%)	32 (38.1%)	16 (19.8%)	0.010
Current smoking, *n*%	22 (13.3%)	13 (15.5%)	9 (11.1%)	0.410
History of CVEs, *n*%	35 (21.2%)	21 (25.0%)	14 (17.3%)	0.226
BMI, kg/m^2^	23.1 ± 3.4	22.9 ± 3.6	23.2 ± 3.1	0.536
Systolic BP, mmHg	150.9 ± 24.6	145.7 ± 26.7	156.2 ± 21.1	0.006
Diastolic BP, mmHg	86.3 ± 15.8	82.2 ± 15.1	90.6 ± 15.4	0.001
Hemoglobin, g/dL	10.1 ± 2.0	10.3 ± 2.0	9.8 ± 1.9	0.082
Creatinine, mg/dL	8.7 ± 2.7	8.0 ± 2.3	9.4 ± 2.9	<0.001
BUN, mg/dL	51.6 ± 19.7	53.8 ± 20.1	49.4 ± 19.2	0.153
Uric acid, mg/dL	6.0 ± 1.7	5.8 ± 1.9	6.1 ± 1.6	0.343
Corrected calcium, mg/dL	10.0 ± 1.1	9.8 ± 1.2	10.2 ± 1.0	0.014
Phosphate, mg/dL	5.1 ± 1.8	5.2 ± 1.8	5.0 ± 1.8	0.461
AKP, IU/L	77 (61.5, 107)	86.0 (66.3, 121.3)	73.0 (58.0, 91.5)	0.010
iPTH, pg/mL	148.1 (59.2, 337.8)	194.7 (62.3, 388.1)	111.4 (55.9, 242.2)	0.039
Total cholesterol, mg/dL	166.8 ± 45.4	168.8 ± 45.9	164.7 ± 45.0	0.561
LDL cholesterol, mg/dL	79.6 ± 29.0	80.7 ± 30.7	78.4 ± 27.1	0.620
HDL cholesterol, mg/dL	38.5 ± 12.1	38.9 ± 11.3	38.0 ± 12.9	0.613
Triglycerides, mg/dL	182.5 (131.5, 239.7)	185.1 (149.9, 238.3)	175.4 (117.4, 241.8)	0.190
CRP, mg/L	4.8 (3.7, 7.4)	4.8 (3.6, 11.3)	4.8 (3.9, 7.0)	0.754
Albumin, g/dL	3.1 ± 0.6	3.2 ± 0.7	2.9 ± 0.5	0.003
Sclerostin, pg/mL	250.9 (163.9, 393.7)	258.1 (163.6, 420.9)	241.4 (163.9, 373.8)	0.878
Active vitamin D use, *n*%	41 (24.9%)	20 (23.8%)	21 (25.9%)	0.753
P binders use, *n*%	23 (13.9%)	10 (11.9%)	13 (16.1%)	0.442
CAC score	22.8 (1.7, 162.3)	28.9 (6.7, 179.7)	16.3 (0, 154.2)	0.086
RRF	0 (0, 3.5)	0 (0, 2.6)	1.6 (0, 4.0)	0.135

HD: hemodialysis; PD: peritoneal dialysis; CVEs: cardiovascular events; BP: blood pressure; BUN: blood urea nitrogen; AKP: alkaline phosphatase; iPTH: intact parathyroid hormone; LDL: low-density lipoprotein; HDL: high-density lipoprotein; CRP: C-reactive protein; CAC: coronary artery calcification; RRF: residual renal function.

Based on the Spearman correlation analysis, the serum sclerostin levels were positively associated with diabetes (*p* = 0.004), but negatively associated with dialysis duration (*p* = 0.036), diastolic BP (*p* = 0.002), and iPTH (*p* = 0.037) in the HD group. Additionally, serum sclerostin levels were positively associated with age (*p* < 0.001), gender (*p* = 0.031), diabetes (*p* = 0.004), and history of CVEs (*p* = 0.022), but negatively associated with diastolic BP (*p* = 0.045) in the PD group. Serum sclerostin levels were not associated with CAC scores in either patients with HD or PD (*p* > 0.05, Supplementary Table S1).

In multivariate linear regression models, the serum sclerostin levels correlated positively with diabetes (*p* = 0.035), but correlated negatively with diastolic BP (*p* = 0.012) in the HD group (Table 2). Besides, the serum sclerostin levels correlated positively with age (*p* = 0.001) and gender (*p* = 0.012) in the PD group (Table 2).

**Table 2.** Multivariate linear regression analyses of the associations between serum sclerostin levels and baseline characteristics in the 165 dialysis patients.

Variable	HD (*n* = 84)		PD (*n* = 81)	
	β (95% CI)	*P*	β (95% CI)	*P* Value
Age	–	–	0.365 (0.154, 0.532)	0.001
Gender	–	–	0.259 (0.052, 0.416)	0.012
Dialysis duration	–	0.108	–	–
Diabetes	0.227 (0.016, 0.442)	0.035	–	0.105
History of CVEs	–	–	–	0.074
Diastolic BP	−0.272 (−0.547, −0.069)	0.012	–	0.905
iPTH	–	0.555	–	–
RRF	–	0.138	–	0.498

HD: hemodialysis; PD: peritoneal dialysis; CI: confidence interval; CVEs: cardiovascular events; BP: blood pressure; iPTH: intact parathyroid hormone; RRF: residual renal function.

### Sclerostin with all-cause mortality and CVEs

During the median follow-up period of 24.9 months (range, 19.4–30.7 months), 27 patients (16.4%) died and 32 patients (19.4%) developed new onset CVEs. The median serum sclerostin level at baseline was 250.9 pg/mL, and we divided all participants into two groups (low serum sclerostin group [group 1, serum sclerostin level ≤250.9 pg/mL] and high serum sclerostin group [group 2, serum sclerostin level >250.9 pg/mL]).

**Figure 1.**
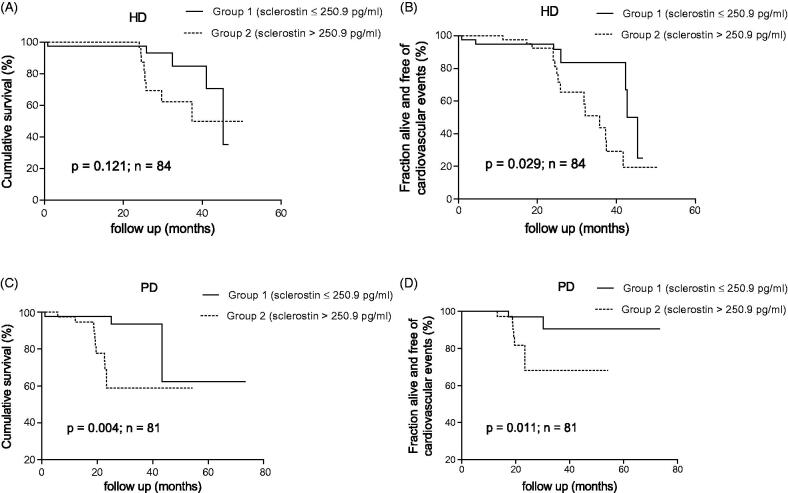
Kaplan–Meier estimates of cumulative survival and CVEs of the 84 HD (A,B) and 81 PD (C,D) patients according to serum sclerostin levels above and below the median.

To determine the relationship between serum sclerostin levels on all-cause mortality and new onset CVEs, the Kaplan–Meier method was used to estimate the cumulative survival rate as well as fraction alive without CVEs. In patients with HD, there was no difference in overall survival rates between groups (*p* = 0.121; Figure 1(A)); the CVE-free survival rates were clearly lower in group 2 compared to group 1 (*p* = 0.029; Figure 1(B)). In patients with PD, both overall and CVE-free survival rates were significantly lower in group 2 than in group 1 (*p* < 0.05; Figure 1(C,D)).

Cox proportional hazard regression models were used to determine whether sclerostin is an independent risk factor for all-cause mortality and CVEs. The crude and adjusted hazard ratios (HRs) for all-cause mortality and CVEs are presented in Tables 3 and 4. In the HD group, serum sclerostin levels were not associated with all-cause mortality or CVEs (Tables 3 and 4). However, in the PD group, serum sclerostin levels were consistently associated with all-cause mortality and CVEs based on the adjusted models (Tables 3 and 4).

**Table 3.** Multivariate Cox regression analysis of risk factors for all-cause mortality in the 84 HD and 81 patients with PD.

	HD		PD	
	RR (95% CI)	*p* Value	RR (95% CI)	*p* Value
Unadjusted	1.275 (0.714, 2.275)	0.411	2.570 (1.355, 4.876)	0.004
Model 1^a^	1.074 (0.561, 2.055)	0.829	2.875 (1.323, 6.245)	0.008
Model 2^b^	0.924 (0.381, 2.240)	0.862	2.381 (1.069, 5.302)	0.034
Model 3^c^	0.970 (0.438, 2.148)	0.940	2.983 (1.196, 7.438)	0.019
Model 4^d^	0.967 (0.473, 1.974)	0.926	2.716 (1.238, 5.962)	0.013

^a^ Model 1 was adjusted for age and gender.

^b^ Model 2 was adjusted for age, gender, total and HDL cholesterol, systolic BP, diabetes and smoking.

^c^ Model 3 was adjusted for age, gender, diabetes, CRP, phosphate, BMI, and albumin.

^d^ Model 4 was adjusted for age, gender, and diabetes in patients with HD, and was adjusted for age, gender, AKP, and CAC score in patients with PD.

HD: hemodialysis; PD: peritoneal dialysis; RR: relative risk; CI: confidence interval; HDL: high-density lipoprotein; BP: blood pressure; CRP: C-reactive protein; BMI: body mass index; AKP: alkaline phosphatase; CAC: coronary artery calcification.

**Table 4.** Multivariate Cox regression analysis of risk factors for CVEs in the 84 patients with HD and 81 patients with PD.

	HD		PD	
	RR (95% CI)	*p* Value	RR (95% CI)	*P* Value
Unadjusted	1.301 (0.875, 1.934)	0.193	3.154 (1.349, 7.374)	0.008
Model 1^a^	1.226 (0.817, 1.841)	0.325	4.364 (1.660, 11.471)	0.003
Model 2^b^	1.268 (0.788, 2.040)	0.329	4.726 (1.442, 15.489)	0.010
Model 3^c^	1.330 (0.810, 2.183)	0.260	4.735 (1.671, 13.419)	0.003
Model 4^d^	1.164 (0.742, 1.828)	0.509	3.819 (1.358, 10.740)	0.011

^a^Model 1 was adjusted for age and gender.

^b^Model 2 was adjusted for age, gender, total and HDL cholesterol, systolic BP, diabetes, and smoking.

^c^Model 3 was adjusted for age, gender, diabetes, CRP, phosphate, BMI, and albumin.

^d^Model 4 was adjusted for age, gender and iPTH in patients with HD, and was adjusted for age, gender, diabetes, iPTH, and CRP in patients with PD.

CVEs: cardiovascular events; HD: hemodialysis; PD: peritoneal dialysis; RR: relative risk; CI: confidence interval; HDL: high-density lipoprotein; BP: blood pressure; CRP: C-reactive protein; BMI: body mass index; iPTH: intact parathyroid hormone.

## Discussion

The major findings of our study were that a low serum sclerostin level appears to predict better overall survival and lower prevalence of CVEs in patients with PD but is not associated with overall survival or CVEs rate in patients with HD. Furthermore, we show that serum sclerostin level was not correlated with CACs in either HD or PD groups.

Our study showed an association between high serum sclerostin level and low survival rate in patients with PD, but no association between sclerostin and survival in patients with HD. Although sclerostin has been associated with bone turnover [10], there has been no confirmed relationship between serum sclerostin and clinical outcome. For example, elevated circulating sclerostin levels have been reported to predict better outcomes [17,21], worse outcomes [18,22], or no relationship with outcomes [23,24] in HD or CKD patients. The conflicting results could be secondary to the use of different sclerostin assays, different populations, and variable dialysis treatment modalities. In our present study, the concentrations of serum sclerostin are similar to the study by Stavrinou et al. [25] using R&D ELISA kits in dialysis subjects. Unlike previous studies, which included only patients with HD or a small cohort of patients with PD, we enrolled nearly equal numbers of patients with HD and PD. Our study found that the relationships between serum sclerostin and mortality were different in variable dialysis treatment modalities. As previously reported, bone turnover was lower, and mineralization was impaired in the PD group compared with the HD group [26]. Our results also showed that AKP and iPTH were significantly lower in patients with PD compared to patients with HD. This could be a plausible explanation for the divergent results of serum sclerostin and mortality between HD and PD populations. Our study also inquired about the use of phosphate binders and vitamin D supplementation, as well as cardiovascular comorbidities, as compared with other studies, which might play a prominent role in influencing mortality and could thus explain, in part, why our study is inconsistent with other published literature. The question whether serum sclerostin has a protective or deleterious role in survival for dialysis patients is still open and needs to be answered.

Our observation that a high serum sclerostin level was associated with a high prevalence of CVEs in patients with PD, but no association between sclerostin and CVEs in patients with HD. The results suggest that there was also a protective effect of low serum sclerostin level for CVE-free survival in patients with PD. Gong et al. [27] also showed the positive link observed between sclerostin and CVEs/cardiovascular mortality. With regard to patients with HD, Kanbay et al. [23] and Kundakci et al. [28] found that serum sclerostin levels were not significantly associated with CVE risk in patients with HD, which is consistent with our findings. Why did we get completely different results in PD and patients with HD? In our study, iPTH levels were much lower in patients with PD compared with patients with HD. Serum sclerostin level was independently associated with pulse wave velocity only in patients without hyperparathyroidism [29]. Elevated pulse wave velocity, a marker of arterial stiffness, can predict CVEs [30]. This may be a possible explanation why sclerostin was associated with CVEs only in patients with PD. Although our study was supported by these studies, further largescale clinical studies with long-term follow-up are needed to determine sclerostin’s role on CVEs in dialysis patients.

In this study, we did not find any evidence that confirmed a relationship between sclerostin and CACs in dialysis patients. Certain studies obtained similar results as ours between circulating sclerostin levels and VC scores in HD and CKD patients [23,31], but other groups reported a negative or positive correlation [14,15,32]. Our findings are in agreement with Brandenburg et al.’ study [31], who reported no association between the amount of CAC and serum sclerostin levels in patients with HD; however, Qureshi et al. [32], Pelletier et al. [33], and Lee et al. [34] all reported that high serum sclerostin levels were associated with the extent of VCs in ESRD patients. In contrast, Wang et al. [35] reported that serum sclerostin levels were lower when AACs were moderate-to-severe in CKD3–5D. This discordance might be attributed to diverse study populations, grouping criteria, and calcification grading systems. Therefore, the true relationship between VCs and sclerostin remains unknown. Future research is urgently needed to determine the true relationship between VCs and sclerostin in dialysis patients.

This study has a few limitations which warrant discussion. First is the relatively small sample size, and that this study was performed at a single center. Also, we performed no other tests of other bone metabolism parameters, such as FGF23, klotho, or vitamin D. In addition, small CACs are likely to be omitted when diagnosed by chest CT. Our data must be confirmed in larger dialysis populations with a longer follow-up time.

## Conclusion

In conclusion, our study showed that low serum sclerostin was associated with better overall survival and lower prevalence of CVEs in patients with PD, but that sclerostin played no role in patients with HD. Furthermore, serum sclerostin level was not correlated with the extent of CACs in dialysis patients.

## Supplementary Material

Supplemental Material
