# Supplementary material for: Association of sclerostin with cardiovascular events and mortality in dialysis patients
Source: Ren Fail. 2020 Mar 26;42(1):282–8. doi: 10.1080/0886022X.2020.1741386 (PMC7170300; doi:10.1080/0886022X.2020.1741386)
Supplement: Supplemental Material [file IRNF_A_1741386_SM5088.pdf]

Table S1. Spearman correlations of baseline characteristics with serum sclerostin levels in the 165 dialysis patients.

Variable	HD (n = 84)		PD (n = 81)	
	r	p	*r*	p
Age	0.128	0.246	0.429	<0.001
Gender (male)	0.095	0.391	0.240	0.031
Dialysis duration	-0.229	0.036	-0.039	0.731
Diabetes	0.311	0.004	0.317	0.004
Current smoking	0.059	0.594	0.020	0.858
History of CVEs	0.149	0.176	0.254	0.022
BMI	0.164	0.135	0.155	0.168
Systolic BP	-0.172	0.117	-0.088	0.434
Diastolic BP	-0.326	0.002	-0.224	0.045
Hemoglobin	0.108	0.328	0.001	0.994
Creatinine	0.035	0.754	0.064	0.570
BUN	-0.092	0.407	0.093	0.409
Uric acid	-0.084	0.450	0.144	0.200
Corrected calcium	0.102	0.354	-0.023	0.839
Phosphate	-0.126	0.253	0.095	0.397
AKP	0.064	0.562	0.084	0.456
iPTH	-0.227	0.037	-0.042	0.713
CRP	0.171	0.119	0.166	0.138
Albumin	0.048	0.665	-0.039	0.731
Active Vitamin D use	-0.047	0.673	-0.045	0.689
P binders use	-0.073	0.511	0.070	0.532
CAC score	0.075	0.498	-0.022	0.842
RRF	-0.212	0.053	-0.208	0.062

HD: hemodialysis; PD: peritoneal dialysis; CVEs: cardiovascular events;
BMI, body mass index; BP: blood pressure; BUN: blood urea nitrogen;
AKP: alkaline phosphatase; iPTH: intact parathyroid hormone; CRP: C-reactive protein;
CAC: coronary artery calcification; RRF: residual renal function.
